# Glucose and lipid assessment in patients with acute stroke

**DOI:** 10.1186/1755-7682-7-45

**Published:** 2014-10-23

**Authors:** Anthonia O Ogbera, Olajumoke O Oshinaike, Olusola Dada, Ayodeji Brodie-Mends, Chukwuma Ekpebegh

**Affiliations:** Department of Medicine, Lagos State University College of Medicine, Ikeja, Lagos, Nigeria; Department of Internal Medicine, Faculty of Health Sciences, Walter Sisulu University, Mthatha, Eastern Cape Province South Africa

## Abstract

**Background:**

Stroke is a major health issue in Nigeria and it is also a common cause of emergency admissions. Stroke often results in increased morbidity, mortality and reduced quality of life in people thus affected. The risk factors for stroke include metabolic abnormalities such as dyslipidaemia and diabetes mellitus (DM). The stress of an acute stroke may present with hyperglycaemia and in persons without a prior history of DM, may be a pointer to stress hyperglycaemia or undiagnosed DM.

**Methodology:**

This was a cross sectional study carried out over a period of one year in a teaching hospital in Lagos, Nigeria. Patients with acute stroke admitted to the hospital within three days of the episode of stroke and who met other inclusion criteria for the Study were consecutively recruited. Clinically relevant data was documented and biochemical assessments were carried out within three days of hospitalization. Tests for lipid profile, glycosylated haemoglobin(HbA1c), and blood glucose at presentation were carried out. The presence of past history of DM, undiagnosed DM, stress hyperglycaemia and abnormal lipid profile were noted. Students t test and Chi square were the statistical tests employed.

**Results:**

A total of 137 persons with stroke were recruited of which 107 (76%) met the defining criteria for ischaemic stroke. The mean age and age range of the Study subjects were 62.2 (11.7) and 26–89 years respectively. The Study subjects were classified according to their glycaemic status into the following categories viz; stress hyperglycaemia, euglycaemia, DM and previously undiagnosed DM. Stress hyperglycaemia occurred commonly in the fifth decade of life and its incidence was comparable between those with cerebral and haemorrhagic stroke. The commonly occurring lipid abnormalities were elevated LDL-C and low HDL.

**Conclusions:**

The detection of abnormal metabolic milieu is a window of opportunity for aggressive management in persons with stroke as this will improve outcome. Routine screening for hyperglycaemia in persons with stroke using glycosylated haemoglobin tests and blood glucose may uncover previously undiagnosed DM.

## Background

Stroke is a growing disease and is the third common cause of death in the world after coronary heart disease and cancer especially in the elderly [1–3]. In Nigeria, Stroke constitutes about 2.4% of all emergency admissions with cerebral infarction making up 49% of all cases [4]. The burden of Stroke in Nigeria is such that it is responsible for 1.8% of all deaths in the emergency unit with case fatality rates that increase from 9% to 46% depending on the duration of the event [4].

Detection of metabolic abnormalities is imperative in persons presenting with acute Stroke as outcomes may be affected by the presence of these abnormalities especially when left untreated. Some metabolic abnormalities that are oft documented in Stroke patients include dyslipidaemia and hyperglycaemia. Non-diabetic patients presenting with an acute stroke often have hyperglycaemia and this may be transient and due to the acute stress response or truly representative of undiagnosed abnormal glucose metabolism. Some Studies have reported higher mean admission glucose levels in non survivors of stroke compared with survivors and thus the importance of screening for this all-important metabolic abnormality in stroke patients cannot be overemphasized [5–7]. Transient hyperglycaemia or stress -induced hyperglycaemia (SH) during acute illness often occurs in patients without previous evidence of diabetes mellitus. Stress-induced hyperglycemia, typically described as blood glucose concentrations above 200 mg/dl, has been described in the literature for almost 150 years [5]. Acute hyperglycemia has been documented to be a predictor of in-hospital mortality after ischemic stroke in nondiabetic patients and increased risk of poor functional recovery in non-diabetic stroke survivors [8].

Possible reasons for the aforestated scenario include direct toxicity of hyperglycaemia to the brain and the potential neurotoxic effect of acidosis which results from anaerobic cerebra lglucose metabolism [8]. Other than stress hyperglycaemia and poorly controlled preexisting diabetes mellitus accounting for elevated blood glucose in Stroke, elevated blood glucose levels may reflect undiagnosed diabetes mellitus [9]. The majority of acute stroke patients have disorders of glucose metabolism, and in most cases this fact has been unrecognized. A diagnosis of diabetes in a stroke patient would not only probably change the clinical management of that patient, specifically with respect to lipid and blood pressure management but also lead to possible institution of insulin administration. This is because intensive insulin therapy not only controls elevated glucose levels in the critically ill, but has been shown to significantly improve outcomes among hospital in patients with acute hyperglycaemia or newly diagnosed diabetes.

Dyslipidemia, defined as elevated total or low-density lipoprotein (LDL) cholesterol levels, or low levels of high-density lipoprotein (HDL) cholesterol, high total cholesterol (TCHOL) and elevated triglyceride (TG) is an important risk factor for coronary heart disease (CHD) and stroke. In persons living with DM, dyslipidaemia is not only a potential independent risk factor for cerebrovascular disease but unfortunately diabetic patients with ischemic stroke remain uncontrolled for dyslipidaemia [10]. In a Cross sectional Nigerian Report [11], that evaluated for lipid parameters in patients with first ever Stroke, serum triglyceride was the lipid parameter that was documented to confer significant stroke risk.

The occurrence of hyperglycaemia presenting as stress hyperglycaemia or new diagnosis of DM in non diabetic patients with stroke is not widely studied in our neck of the woods.

The main Objective of this Study is the documentation of the frequency of occurrence of Stress hyperglycaemia, undiagnosed DM, DM and dyslipidaemia in patients with acute Stroke.

### Methodology

This was a cross sectional study carried out in patients presenting with Stroke to the Emergency Unit of the Lagos State Teaching Hospital, Ikeja, Lagos from January 2012 to December 2013.The inclusion criteria are as stated below; Patients > 18 years of age hospitalized with a sudden neurologic deficit consistent with cerebral infarction, ischemia or hemorrhage.

Exclusion criteria included patients with a neurologic deficit attributable to neoplasm, trauma, or subdural or epidural hematoma and patients whose symptoms resolved <24 hours after admission. Patients with hepatic diseases, renal diseases, malignancies and those who were on lipid lowering medications or on Steroids prior to the onset of Stroke were also excluded.

### Clinical and biochemical assessment

At presentation, the blood pressure, pulse rate, and capillary random blood glucose levels were documented. (These data were obtained within 24 hours of admission).

Interviewer administered questionnaires were used to capture the following data; age, sex, body mass index, history of diabetes mellitus, hypertension, smoking and hyperlipidemia. The biochemical parameters that were evaluated for included random capillary glucose, and plasma glycosylated haemoglobin (HbA1c), fasting total serum cholesterol, low density lipoprotein (LDL) cholesterol and high density lipoprotein (HDL). Samples for biochemical analyses were taken within 72 hours of hospitalization. HbA1c was assayed using a fully automated Boronate Affinity assay for the determination of the percentage of hemoglobin A1C (HbA1c%) in whole blood. A confirmed value of HbA1c at or above 48 mmol/mol (6.5%) is used as diagnostic for diabetes mellitus as recommended by the American Diabetes Association [12].

The diagnosis of Stroke was made clinically and with the aid of Computed tomographic scans for some patients. Brain CT Scans were carried out in most of the patients and delineated between ischaemic and haemorrhagic stroke. (Imaging results were given by the Consultant Radiologists of the hospital). However where imaging was not done clinical criteria were used to differentiate between haemorrhagic and ischaemic stroke.

Ethical approval was obtained from the Lagos State University Teaching Hospital Ethics Committee and informed consent was obtained from the study subjects or relations.

### Operational definitions

1. Stroke is defined as rapidly developed clinical signs of focal (or global) disturbance of cerebral function lasting more than 24 hours (except in cases of sudden death or if the development of symptoms is interrupted by a surgical intervention), with no apparent cause other than a vascular origin [13]. The siriraj stroke score was used in the assessment and diagnosis of stroke cases in the study. Earlier studies had shown that the siriraj stroke score correlated significantly with CT diagnosis and as such recommended for use in settings where CT is not available or affordable [14].
2. Stress hyperglycaemia in persons without evidence of previous diabetes mellitus refers to random blood glucose >11 · 1 mmol/L in the presence of glycosylated hameoglobin levels of <6.5%.
3. Hypertension: Individuals with raised arterial pressure ≥140/90 or patients on antihypertensive medication.
4. Dyslipidaemia: Abnormal lipid profile consists of the following abnormalities either singly or in combination. These include triglyceride (TG) levels ≥ 150 mg%, high density lipoprotein cholesterol (HDL-C) (for men ≤ 40 mg% and women ≤ 50 mg%), low density lipoprotein cholesterol (LDL-C) ≥ 100 mg% [11].
5. The diagnosis of “undiagnosed diabetes mellitus” was made if patients with no prior history of DM had a random glucose >11.1 mmol/L showed an admission HbA1c >6.5% [11].
6. Prior history of DM/Known DM refer to persons who were already on glucose lowering agents before the stroke episode.

Statistical analysis was performed using Chi-square for qualitative variables and Students t test for quantitative data. P value of 0.05 or less was considered as the level of significance.

We classified the patients into four categories viz, euglycaemia with no history of DM, history of DM, hyperglycaemia with no prior history of DM which was further classified into stress hyperglycaemia and undiagnosed diabetes mellitus.

## Results

There were 137 patients with Stroke (the diagnosis of Stroke was made via CT scanning in 72 persons). The mean age and age range were 62.2(11.7) and 26–89 years respectively. The male subjects were 80 in number and the females who were 57 made up 42% of the Study subjects. The mean age of the female subjects was higher than that of the males but this difference was not statistically significant (63.14(11.6) Vs 61.5(11.9), p = 0.4. The patients who had ischaemic Stroke were 104 in number and made up 76% of the Study population. The mean age (standard deviation) of persons with ischaemic Stroke was comparable to that of persons with haemorrhagic Stroke (62.3 (11.7) years Vs 60.3 (11.8) years, p-0.6).

Less than a third of the Study subjects had a prior history diabetes mellitus. This and other baseline features of the Study population are shown in Table 1.

**Table 1:** **Base line characteristics of Stroke patients**

Parameter	No (frequency)
Males: females	80(58%): 57(42%)
History of diabetes mellitus	33(24%)
Ischaemic CVA	104(76%)
Haemorrhagic CVA	33(24%)
Repeat CVA	32(23%)

### Biochemical parameters

#### Lipid parameters

The mean values of plasma total cholesterol (181.23 vs 185.45 mmol/L, P = 0.3), low-density lipoprotein cholesterol (118.12 vs 125.09, p = 0.4), and high-density lipoprotein (43.62 vs 44.27, P = 0.1) were comparable between persons with ischaemic Stroke and those with haemorrhagic Stroke. The mean serum triglyceride level was higher in persons with ischaemic Stroke compared to that of persons with haemorrhagic Stroke. (95.49 vs 88.39 p = .04) and this difference was statistically significant. Elevated LDL-C (57%) and reduced HDL-C (48%) were the commonly documented lipid abnormalities in the Study population. The proportions of patients with elevated total cholesterol and hypertriglyceridemia were 29% and 13% respectively.

#### Glycaemic parameters

Persons with a history of diabetes mellitus were 33 and made up 24% of the Study population.

**Figure 1:**
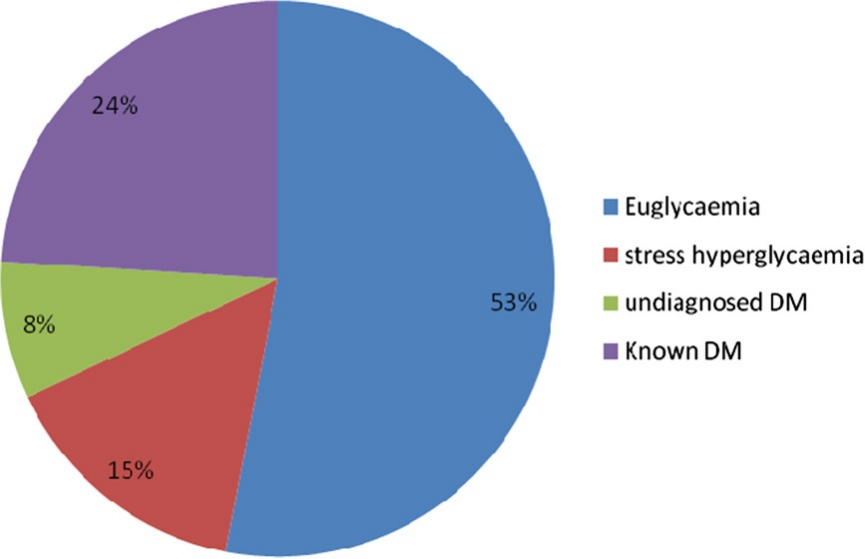
**Distribution of stroke patients according to their glycaemic status.**

**Figure 2:**
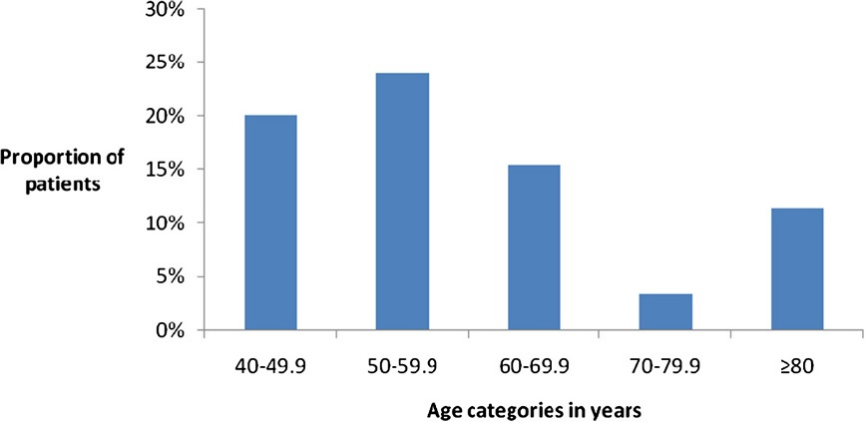
**Age distribution of stress hyperglycaemia in patients with stroke.**

Undiagnosed DM was noted in 11 of the patients hence the frequency of occurrence of DM in the Study population was 39%. The diagnosis of Stress hyperglycaemia was made in a total of 20 patients of which the proportions of this parameter in persons with ischaemic Stroke and those with haemorrhagic Stroke was comparable (15% Vs 12%, p = 0.6). About half of the Study subjects were euglycaemic and this result is shown in Figure 1. The mean age of persons with Stress hyperglycaemia was lower than that of persons without stress hyperglycaemia but this difference was not statistically significant (63 Vs 58, p = 0.9). Stress hyperglycaemia was noted most commonly in the fifth decades of life. These results are shown in Figure 2.

## Discussion

This Report shows that hyperglycaemia is a common feature of acute stroke, occuring in 23% of persons with no prior history of diabetes mellitus. Stress hyperglycaemia in the context of this study accounted for 68% of the persons with hyperglycaemia. We also note that the occurrence of stress hyperglycaemia in persons with haemorrhagic stroke was comparable to that of those with ischaemic stroke.

Although our results show that the mean age of the Study subjects with stress hyperglycaemia, was lower than that of the subjects without stress hyperglycaemia, the fifth decade of life was the commonest age decade where stress hyperglycaemia was documented. Our results differ from that of Al-Azzawi who noted that stress hyperglycaemia was commonly documented in the seventh decade of life [15]. It is pertinent to note that the age range of persons with stroke in this report -26-89 years-is similar to that by Al-Azzawi et al. -27 -84 years and that stress hyperglycaemia was not present in persons less than 40 years of age in both reports. The adverse effects of stress hyperglycaemia have been widely studied and the mechanisms responsible for this all important prognostic factor of stroke include the release of cortisol and norepinephrine [16]. Stress hyperglycemia may also be a marker of deficient glucose regulation in individual with insulin resistance and developing diabetes mellitus [17].

The prevalence of recognized diabetes mellitus in acute stroke patients is between 8% and 20%, but between 6% and 42% of patients may have undiagnosed diabetes mellitus before presentation ([6]). The prevalence of undiagnosed DM in this Report -8%- falls within the aforestated range but slightly lower than in the earlier stated Report by Al-Azzawi et al. [15]. However a higher incidence of undiagnosed DM in stroke -16.7%- was noted in a Japanese Report [18] but this may be ascribed to the fact that oral glucose tolerance test was employed in making the diagnosis. In our report, the mean values of plasma total cholesterol, low-density lipoprotein cholesterol, and high-density lipoprotein were comparable between persons with ischaemic Stroke and those with haemorrhagic Stroke. We note in this study that the mean levels of triglyceride were higher in patients with Ischaemic stroke compared with that of haemorrhagic stroke. Our results show that the pattern of lipid abnormalities in persons with stroke in decreasing order of occurrence are high LDL, low HDL, high TCHOL and high TG.

From the foregoing we have showed that opportunistic screening for hyperglycemia in persons presenting with stroke may unmask not only stress hyperglycaemia but undiagnosed DM. Stress hyperglycaemia occurs as a response to the stress of acute stroke but undiagnosed DM is a risk factor for stroke and if not discovered puts the patients at risk for not only poor outcomes but also for the development of a repeat Stroke.

Dyslipidaemia is a notable risk factor for cardiovascular diseases and its association with the incidence of acute stroke is well documented [19]. The levels of the lipid parameters TC, LDL-C, and HDL-C have also been shown to be related with the outcome in stroke patients [20]. Unlike in our findings, an Indian Report noted that the proportion of patients with ischaemic stroke with lipid abnormalities was much higher than those with haemorrhagic stroke and lipid abnormalities [21]. The implication of these findings are not clearly defined.

The diagnosis of diabetes mellitus (DM) has, until recently, been based on blood glucose levels, i.e. either fasting plasma glucose (FPG) ≥7.0 mmol/l or an oral glucose tolerance test (OGTT) result of ≥11.1 mmol/l [22]. The International Expert Committee of Diabetes, American Diabetes Association and the World Health Organization (WHO) have included HbA1c value ≥ 48 mmol/mol (6.5%) as an additional method for diagnosing DM [23]. About 60% of our patients without a prior history of DM who had admission hyperglycaemia had normal HbA1c concentrations. Since the HbA1c levels provide an indication of the average blood glucose concentration during the preceding 3 months, this observation would imply that fasting hyperglycaemia may reflect undiagnosed diabetes in some patients, while in others it is a reflection of an acute stress response to cerebral damage. Most studies that investigated the use of HbA1c values against OGTT as a diagnostic tool for DM have found reduced prevalence by HbA1c criteria compared with the OGTT criteria [24]. Oral glucose tolerance test is thus a far more sensitive and reliable test of diabetes mellitus than fasting blood glucose or HbA1c. We are of the opinion that the yield of undiagnosed DM in this report may have been higher if an OGTT test was administered instead of the HbA1c test. The OGTT test is however not convenient for administration to acutely ill stroke patients and researchers often administer the test post the acute phase of the stroke episode.

We suggest that all patients admitted for stroke and without a prior diagnosis of DM should be screened for identification of those with abnormal glucose metabolism. Lipid profile should also be checked in acute stroke although the exact timing of the tests is not exactly known.

## Conclusion

The detection of abnormal metabolic milieu in persons presenting with stroke makes a case for aggressive treatment to improve stroke outcomes and secondary prevention of further cerebrovascular disease.

### Limitations of the study

The results of the Study should be interpreted with caution as CT scan was not carried out in all patients.
